# Simultaneous Atomic Resolution Imaging and Electronic Characterization of Wet-Chemically Prepared Nanocrystals

**DOI:** 10.1021/acs.nanolett.6c01858

**Published:** 2026-07-10

**Authors:** Auke Vlasblom, Victor Wesselingh, Jara Vliem, Daniel Vanmaekelbergh, Ingmar Swart

**Affiliations:** Debye Institute for Nanomaterials Science, Faculty of Science, Utrecht University, Princetonplein 1, Utrecht, 3584 CC, The Netherlands

**Keywords:** 2D materials, Bi_2_Se_3_, nanoplatelets, point defects, scanning tunneling microscopy

## Abstract

Wet-chemical colloidal synthesis has emerged as a major platform for producing metallic, magnetic, and semiconducting nanomaterials, which have extensive applications in optoelectronics and hold promise in quantum technology. However, simultaneous characterization of the atomic and electronic structure—correlating the two—has not been possible for these materials due to the detrimental effects of surface ligands and solvent molecules. Using a viscoelastic transfer method, we could deposit isolated, wet-chemically prepared Bi_2_Se_3_ nanoplatelets onto an atomically clean surface in ultrahigh vacuum, with limited cotransfer of solvent molecules. We image the atomic structure of the surface with scanning tunnelling microscopy and characterize the impact of (sub)­surface point defects and adsorbates on the electronic band structure. This work opens the stage to connect the atomic structure to the electronic and optical properties of wet-chemically prepared nanocrystals—a long-standing challenge for one of the major platforms in materials science.

## Graphical abstract

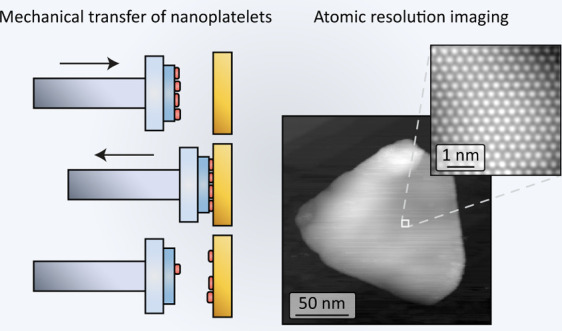

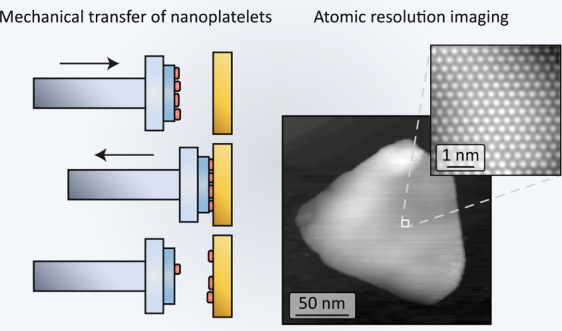

At present, wet-chemical colloidal synthesis allows control over the size, shape, and surface termination of nanocrystals close to atomic precision.^1,2^ This level of control has boosted the widespread use of wet-chemically prepared nanocrystals in numerous optical applications.^3^ Understanding how the electronic structure relates to the size, shape, and atomic structure of a nanocrystal, including point defects and surface termination, is a crucial element of spectral engineering. An important effort employs (scanning) transmission electron microscopy ((S)­TEM) to connect the atomic structure of a single nanocrystal to its photoluminescence properties.^4,5^ However, it remains challenging to simultaneously characterize the atomic structure of the surface and atomic-scale defects, let alone study the impact of a single defect.

Another major question is how the properties of nanocrystals differ from those of their bulk counterparts.^6^ This relates, among others, to the large fraction of atoms at the surface of nanocrystals, often involving a variety of facets and surface terminations. Second, bulk materials and nanocrystals are usually synthesized using different methods, possibly resulting in different crystalline quality (e.g., regarding the number and type of defects). Obtaining information about the number and types of defects present in wet-chemically prepared nanocrystals is therefore an important but largely outstanding problem.

Relating the atomic structure to the electronic structure on the local scale is possible with scanning tunnelling microscopy (STM) and spectroscopy (STS). This has been achieved for technically relevant semiconductors,^7–11^ magnetic materials,^12^ and superconductors^13^ that are prepared in an ultrahigh vacuum environment. With STM, achieving atomic resolution and simultaneously measuring the electronic structure has been achieved for *in situ* prepared samples, such as those that are exfoliated in vacuum,^14^ grown via molecular-beam epitaxy (MBE),^15^ or prepared via transfer methods.^16^ In contrast, wet-chemically prepared nanocrystals could so far not be imaged with atomic-scale spatial resolution with STM.^17^ The challenge lies in the existing sample preparation methods that cannot sufficiently remove solvent molecules and/or organic ligand stabilizers that remain absorbed on the nanocrystal surface after deposition. These molecules and ligands prevent direct access to the surface of the nanocrystal and may adsorb on the tip apex, which can lead to instabilities in the tip–sample junction and, consequently, prevent atomic-scale imaging. Depending on the ligand, they may also influence the electronic structure, e.g., via charge transfer.

The transfer of wet-chemically prepared nanocrystals onto a substrate unavoidably involves a wet transfer step. Nanocrystals can be directly drop-cast onto a substrate, but this results in unwanted solvent molecules that adsorb on the substrate and nanocrystal surface, preventing atomic resolution imaging.^17,18^ Therefore, a two-step process is required in which nanocrystals are first drop-cast on a stamp that, in a second step, can be brought into ultrahigh vacuum and pushed against an ultraclean substrate to mechanically transfer the nanocrystals.^16,19–24^

In this work, we use such a multistep technique, coined viscoelastic nanoparticle transfer (VENT), to demonstrate the simultaneous imaging and characterization of (partially) atomically clean wet-chemically prepared nanoparticles. We use two-dimensional Bi_2_Se_3_ nanoplatelets deposited on Au(111) as an example. We can image the nanocrystal surface with atomic resolution, including surface and subsurface point defects of any given nanoplatelet and simultaneously measure its local density of states. We find that some (sub)­surface point defects induce electronic doping (v_Se(1)_, Se_Bi(2)_, and Bi_Se(3)_), while other defects impose electronic states close to the Fermi level (X_Se(1)_ and Bi_Se(1)_). In addition, we show that adsorbates play an important role in the local electronic structure of the nanoplatelet surface: The local dipole of the adsorbates locally lowers the work function of the nanoplatelet surface, which results in a change in the local potential. We hence connect the atomic structure to the electronic properties of wet-chemically prepared nanocrystals—simultaneously measured on a local scale—achieving the same high standards as have been demonstrated for in-vacuum prepared surfaces. Finally, we find clustering of particular types of defects, suggesting local inhomogeneities during the synthesis.

For an STM study on single Bi_2_Se_3_ nanoplatelets, the nanoplatelets need to be isolated from other nanoplatelets, and the coverage needs to be sufficient (the field of view in a low-temperature STM is typically in the order of a μm^2^). Furthermore, the substrate and nanoplatelet surface should be free of solvent molecules, ligands, and other contaminants. These criteria are not met when using traditional methods for nanocrystal-to-substrate transfer, such as drop-casting (see Supplementary Figure 1).^17^ As we will now show, the VENT technique described below results in a sample suitable for STM studies.

A schematic overview of the sample preparation is given in Figure 1a,b. The method is based on existing assembly techniques for nanocrystal transfer,^19,24^ except that the dry transfer of nanocrystals takes place under ultrahigh vacuum conditions and onto an atomically clean substrate.^16^ We are not aware of other dry transfer techniques that take place under ultrahigh vacuum conditions and/or that allow atomic resolution imaging of wet-chemically prepared nanoparticles with STM. In brief, a dry stamp is created by drop-casting a droplet of Bi_2_Se_3_ nanoplatelets on plasma-treated polydimethylsiloxane (PDMS) mounted on a glass slide. PDMS is commonly used for the transfer of 2D materials^19,24^ and the production of devices.^16,25^ An essential step in our procedure is the plasma treatment of the PDMS and glass slide to create strong adhesion between the glass and PDMS,^26^ remove residue from the PDMS surface,^27^ and increase the wettability of the PDMS surface.^28^ After plasma treatment and drop-casting, the nanoplatelet-covered dry stamp is brought into an ultrahigh vacuum chamber, where it is then pressed onto a vacuum-prepared Au(111) surface, resulting in mechanical transfer of nanoplatelets. Au(111) is chosen as the target substrate for two main reasons: this substrate allows *in situ* tip conditioning, and atomic features are easily detected, i.e., herringbone reconstruction and steps, which are useful to assess the substrate surface cleanliness after nanoplatelet transfer.

**Figure 1:**
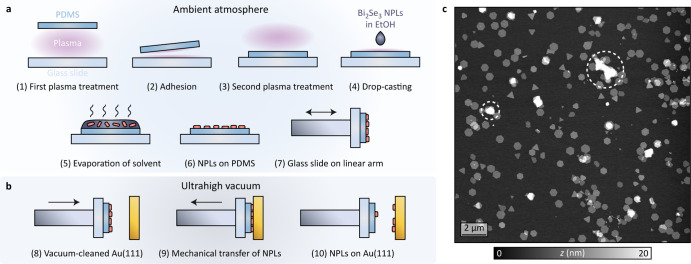
**Overview of the VENT technique. a**,**b**, Schematic overview of the steps of the VENT technique. **a,** First, a stamp is fabricated by drying a droplet of Bi_2_Se_3_ nanoplatelets on a piece of plasma-treated PDMS under ambient conditions. **b**, Subsequently, the stamp is brought into ultrahigh vacuum. Then, the stamp is pressed against a vacuum-prepared Au(111) substrate, resulting in mechanical transfer of nanoplatelets. **c** AFM image of Bi_2_Se_3_ nanoplatelets transferred to mica under ambient conditions with the VENT technique. There is an appropriate surface density of individual nanoplatelets. Only a few regions show stacked nanoplatelets. Two somewhat larger stacks are indicated by dashed white circles.

To illustrate the distribution and coverage of nanoplatelets after deposition, Figure 1c shows a large-scale AFM image of nanoplatelets transferred onto mica under ambient conditions. Many isolated nanoplatelets spread out over the surface can be seen (the coverage is approximately one nanoplatelet per 4 μm^2^). There are only a few stacks of nanoplatelets, indicated by the dashed circles. More details and comments about the transfer procedure can be found in Supplementary Note 1.

Figure 2a shows an STM image of a single Bi_2_Se_3_ nanoplatelet with a thickness of four quintuple layers (see Supplementary Figure 2) transferred to Au(111) using the transfer method described above. By zooming in on the central area of the nanoplatelet, we resolve the atomic structure of the Bi_2_Se_3_(0001) surface (see Figure 2b). We extract a lattice constant *a* of 410 ± 2 pm, which matches the lattice parameter of cleaved bulk Bi_2_Se_3_ crystals found in the literature.^14^

**Figure 2:**
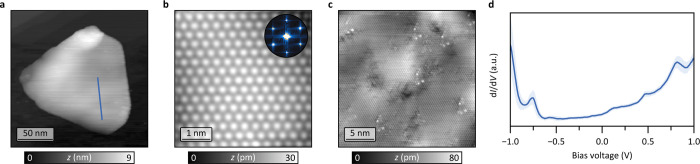
**Characterization of a single Bi**_**2**_ **Se**_**3**_ **nanoplatelet. a**, STM image of a single free-standing four-quintuple-layer nanoplatelet on Au(111) (*V*_bias_ = 1 V, *I*_set_ = 50 pA). Background correction (plane leveling) is applied. **b**, STM image of the interior of a nanoplatelet (*V*_bias_ = −1 V, *I*_set_ = 200 pA). Background correction (plane leveling) and a 2D FFT filter are applied. The inset shows the 2D FFT of the image, showing the hexagonal structure of the Bi_2_Se_3_ surface. **c**, STM image of the interior of the nanoplatelet shown in (a) (*V*_bias_ = 1 V, *I*_set_ = 50 pA). Different types of native point defects can be observed. Polynomial background correction (see Supplementary Figure 7) and a 2D FFT filter are applied. **d**, Average of 60 d*I*/d*V* spectra taken along a 61 nm line on an atomically clean area on the nanoplatelet depicted in (a). The gloom represents the standard deviation.

STS was performed to investigate the electronic structure of this nanoplatelet (see Figure 2d). The d*I*/d*V* spectrum is an average of 60 spectra taken along a 61 nm line on an atomically clean part (the effect of adsorbates on the electronic structure is discussed below) of the surface of the nanoplatelet shown in Figure 2a. The very small standard deviation (blue shade) demonstrates that the electronic structure is spatially homogeneous across the nanoplatelet surface. The spectrum of the nanoplatelet is qualitatively similar to that of a thin Bi_2_Se_3_ film (grown with MBE) with the same number of quintuple layers.^29^ STS on atomically clean and defect-free areas on five different four-quintuple-layer nanoplatelets show similar electronic structures (see Supplementary Figure 3). These five nanoplatelets likely experienced different forces during transfer, and thus have small variations in strain. Yet the Fermi level is similar (a similar degree of *n*-type doping). In addition, strain induced by the stacking of two nanoplatelets does not significantly influence the electronic structure (see Supplementary Figure 4). These observations suggest that either strain (induced by the VENT method) does not strongly affect the electronic structure, or that the VENT method does not induce strain in the nanoplatelets.

A prominent peak is observed in the valence band at –750 mV, together with a series of steps in the conduction band (see Figure 2d). The peak and steps have been attributed to quantum well states of the thin nanoplatelet,^29,30^ as was also observed for another topological insulator.^31^ A major difference between the nanoplatelet and a thin film, however, is the position of the prominent valence band peak. The peak position is reported at –600 mV for a four-quintuple-layer Bi_2_Se_3_ film grown on 6H-SiC(0001), while we observe the peak at –750 mV, suggesting a larger degree of *n*-type doping. We note that the work function of Bi_2_Se_3_ reported in the literature varies (4.7–5.4 eV),^32,33^ and is close to the work function of Au(111) (5.2 eV).^34^ The exact work function of the Bi_2_Se_3_ nanoplatelets is not known, and charge transfer (if present) between the Au(111) substrate and Bi_2_Se_3_ nanoplatelet could result in either a positive or negative spectral shift. We note that *n*-type doping is commonly observed in Bi_2_Se_3_, often showing variations in the degree of *n*-type doping between reports.^29,35–39^ Given that we observe a similar amount of *n*-type doping for five different nanoplatelets with the same thickness (see Supplementary Figure 3), we argue that the doping level we observe is intrinsic and a result of the synthesis conditions.

After nanoparticle transfer, some residue is present on the Au(111) surface (see Supplementary Note 2), but the well-known herringbone reconstruction is still visible. This residue, in addition to the presence of (monatomic) steps in the Au(111) surface, may contribute to the observation that the nanoplatelet shown in Figure 2a displays some corrugations. We also note that not all investigated nanoplatelet surfaces are as atomically clean as the ones shown in Figure 2a–c. The cleanliness varies between different nanoplatelets and different positions on the Au(111) sample. However, the observed coverage of contaminants on the nanoplatelet surfaces was always less than a monolayer (see Supplementary Figure 6a,b), i.e., atomically clean regions were observed on every nanoplatelet that we encountered. In addition, several broken nanoplatelets were found, which were damaged during the mechanical transfer (see Supplementary Figure 6c,d). More than 50 individual nanoplatelets were studied, distributed over seven VENT procedures. Given the slow scanning speed of STM and the necessity of an atomically sharp tip and a stable tip–sample junction, the technique is not well suited to make detailed statistical claims. Given the above, we refrain from making detailed statistical claims on the cleanliness and damage of the nanoplatelets.

The presence of contamination on the Au(111) and nanoplatelet surface, and the occurrence of damaged nanoplatelets, show that there is room for improvement in the transfer procedure (beyond the scope of the current work). Our recommendations include, but are not limited to, the following: optimizing the plasma treatment to reduce contamination, investigating the required amount of pressure during stamping to avoid damaging the nanoplatelets, optimizing the alignment between stamp and target substrate, optimizing annealing, and performing the ambient steps under an inert atmosphere to further improve cleanliness.

A larger-scale atomically resolved image, shown in Figure 2c, immediately reveals different types of native point defects.^14,36,40^ Defects in Bi_2_Se_3_ and Bi_2_Te_3_ have been previously investigated for crystals cleaved in vacuum^14,35–39,41^ and MBE-grown layers.^15,40,42^ However, only a few of these report on STS experiments to study the local electronic effect of single (sub)­surface defects in three-dimensional Bi_2_Se_3_ or Bi_2_Te_3_ crystals.^14,15,35,37–39^ For wet-chemically prepared (2D) nanocrystals, simultaneous topographic and electronic defect characterization has not been performed. Our VENT technique now opens the possibility to simultaneously characterize the atomic and electronic structure of (point) defects that occur in Bi_2_Se_3_ nanoplatelets, and allows us to examine if there are any differences with cleaved crystals or MBE-grown thin films. We identify the dominant defects in Bi_2_Se_3_ nanoplatelets and compare them to those observed for *in situ* prepared single Bi_2_Se_3_ and Bi_2_Te_3_ crystals reported in the literature.^14,35–39,41^

To guide the identification of Bi_2_Se_3_ point defects, a schematic representation of the Bi_2_Se_3_ crystal structure is provided in Figure 3a–c. Each atomic layer is numbered, starting at the surface with Se(1). Three unique sites, labeled A, B, and C, are distinguished as indicated in Figure 3b.^38^ Examples of several point defects in Bi_2_Se_3_ are shown schematically in Figure 3c.

**Figure 3:**
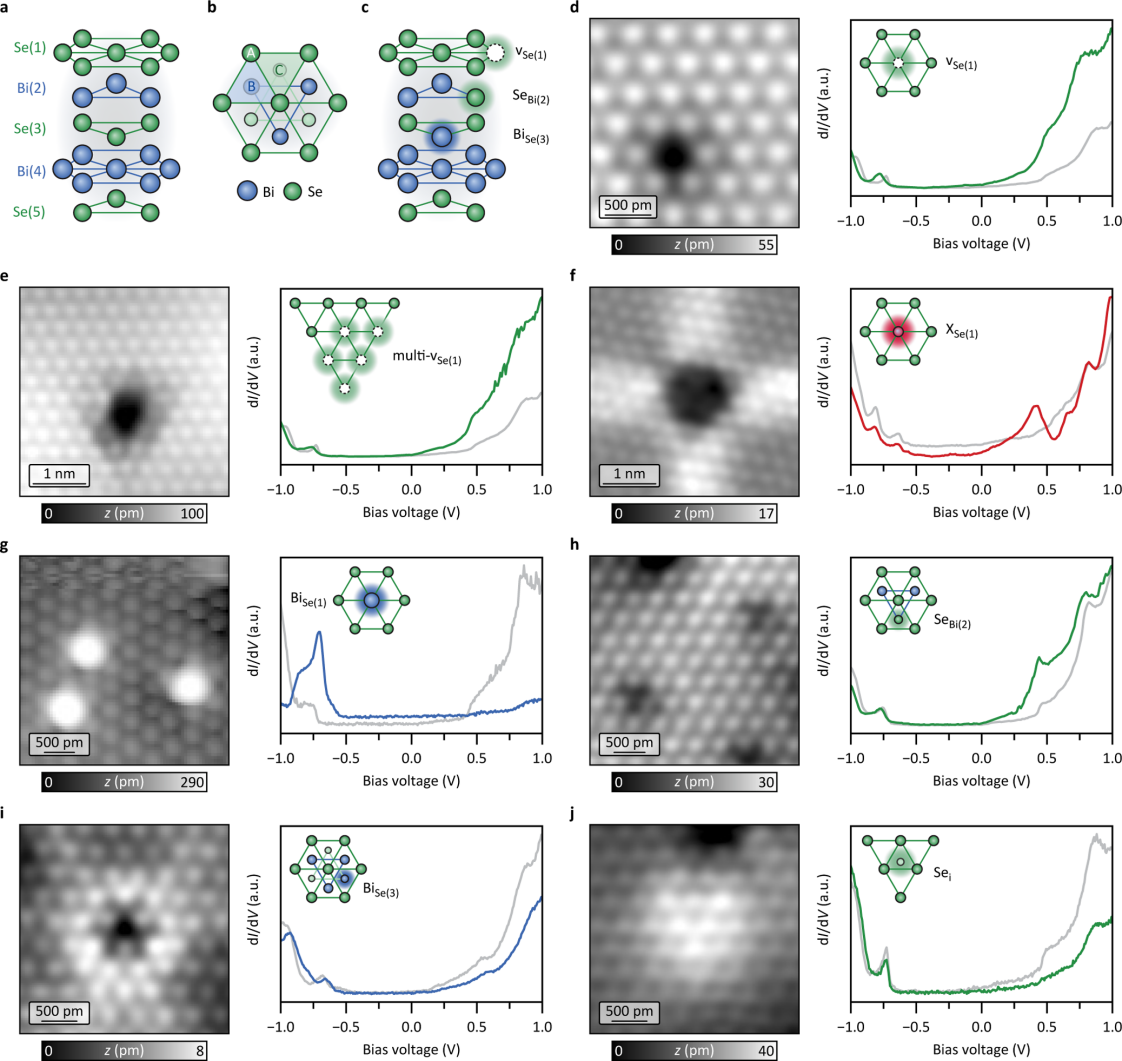
**Topographic and electronic characterization of defects in Bi**_**2**_ **Se**_**3**_ **nanoplatelets. a**, Schematic of a single quintuple layer of the Bi_2_Se_3_ lattice with numbered atomic layers, where the Se(1) layer indicates the surface. **b**, Schematic top view of the Bi_2_Se_3_(0001) lattice. Three unique sites—A, B and C—are distinguished. **c**, Schematic of several point defects. v_Se(1)_ resides at A sites, while Se_Bi(2)_ and Bi_Se(3)_ reside at B and C sites, respectively. **d–j**, STM images (left) of a defect and d*I*/d*V* spectra (right) measured on the defect (colored curve) and away from the defect on an atomically clean and defect-free area (gray curve). All STM images are plane-leveled, except (f), where a polynomial background correction is applied, and 2D FFT filtered (except (g)). **d**, Se(1) vacancy (v_Se(1)_) (*V*_bias_ = −1 V, *I*_set_ = 100 pA). **e**, Multiple Se(1) vacancies (multi-v_Se(1)_) (*V*_bias_ = −0.8 V, *I*_set_ = 100 pA). **f**, Impurity on a Se(1) site (X_Se(1)_) (*V*_bias_ = −0.8 V, *I*_set_ = 400 pA). **g**, Three Bi antisite defects on Se(1) sites (Bi_Se(1)_) (*V*_bias_ = −1 V, *I*_set_ = 50 pA). **h**, Two Se antisite defects on Bi(2) sites (Se_Bi(2)_) (*V*_bias_ = 1 V, *I*_set_ = 0.5 nA). **i**, Bi antisite defect on a Se(3) site (Bi_Se(3)_) (*V*_bias_ = −1 V, *I*_set_ = 40 pA). **j**, Se interstitial defect (Se_i_) located between the first and second quintuple layer (*V*_bias_ = −1 V, *I*_set_ = 100 pA).

Figure 3d–j show an overview of seven different types of defects that were found in multiple Bi_2_Se_3_ nanoplatelets. For each defect, we provide a topographic STM image, along with a d*I*/d*V* spectrum taken directly on the defect (colored curves), and on an atomically clean and defect-free area (gray curves) on the same nanoplatelet. We utilize the position of the prominent peak(s) in the valence band to identify if defects lead to a (positive or negative) shift in the d*I*/d*V* spectra.

First, we identify (multiple) Se(1) vacancies (v_Se(1)_), see Figure 3d,e. Clearly, Figure 3d shows a missing Se(1) atom, which is most easily observed at negative bias.^14^ Figure 3e shows a multi-v_Se(1)_, where five Se(1) atoms are missing (see Supplementary Figure 8). The d*I*/d*V* spectra in Figure 3d,e taken on the (multi)­v_Se(1)_ are shifted to negative energies with respect to the bulk. A single v_Se(1)_ and multi-v_Se(1)_ shift the spectrum by roughly –50 mV and –30 mV, respectively (see Supplementary Figure 9a,b), consistent with v_Se_ being an *n*-type defect.^14,38^ For these type of defects, we observe an increase in conduction band intensity at positive bias—a result that has not been observed in previous reports.^14^ We attribute this to an increased overlap of the tip- and the Bi *p*_*z*_-orbitals. The conduction band of Bi_2_Se_3_ is mainly derived from Bi *p*_*z*_-orbitals.^41^ When a d*I*/d*V* spectrum is taken on (inside) a v_Se(1)_, the STM tip moves closer to the Bi(2) layer, resulting in stronger overlap between tip- and Bi *p*_*z*_-orbitals and thus in a higher conduction band intensity.

Next, we identify a new type of defect in Figure 3f, which has not been reported before for Bi_2_Se_3_ or Bi_2_Te_3_. In the topographic image, there appears to be an indent at a Se(1) site, albeit that the indent appears not as deep as a v_Se(1)_ (see Supplementary Figure 10). Also note that the appearance of the defect in the constant-current topography image is different from that of a v_Se(1)_. The d*I*/d*V* spectrum taken on the indent does not show a shift with respect to the bulk. Instead, a new state appears at 0.4 V. To identify the defect, a constant height d*I*/d*V* map is taken at the energy of the additional resonance and compared to the Se(1) lattice (see Supplementary Figure 11). The d*I*/d*V* map shows localized intensity at a Se(1) site with a triangular symmetry around the defect. We identify this defect as an impurity X at a Se(1) site (X_Se(1)_). Since the defect is localized at a Se(1) site and has a lower apparent height than a Se(1), we propose that the impurity is an iso-electronic element with a smaller radius than Se, such as O, which is present in the Bi and Se precursors used in the synthesis.

In addition to Se vacancies and an impurity defect, we observe three antisite defects: Bi_Se(1)_, Se_Bi(2),_ and Bi_Se(3)_, presented in Figure 3g–i. The three defects reside at A, B, and C sites, respectively, as shown in Supplementary Figure 12. For both Bi_Se(1)_ and Bi_Se(3)_, we observe a shift in the d*I*/d*V* spectra to positive energies compared to the bulk (see Supplementary Figure 9d), which is reminiscent of a *p*-type defect.^14^ Interestingly, we observe resonances in the valence band for Bi_Se(1)_ (see Figure 3g). The shape of the resonances differs between different Bi_Se(1)_ sites and is also observed for a Bi adatom (see Supplementary Figure 13), indicating that this resonance reflects the local symmetry. In addition, we observe a low conduction band intensity. Similar to the previous discussion, we attribute this to reduced overlap between the tip- and Bi *p*_*z*_-orbitals, because the tip is further away from the Bi(2) layer on top of a Bi_Se(1)_ site. In the case of Se_Bi(2)_, we observe a shift toward negative energies for the d*I*/d*V* spectrum taken on the defect (see Supplementary Figure 9c), demonstrating that this defect is an *n*-type defect, which is consistent with previous reports.^14^ The spatial electronic structure of the Bi_Se(3)_ is elucidated with constant height d*I*/d*V* maps, showing a clover-leaf shape (see Supplementary Figure 14).

Last, we present a Se interstitial (Se_i_) residing between the first and second quintuple layer (see Figure 3j).^36,38^ The topographic image shows a small triangular protrusion. The triangular symmetry is also established in d*I*/d*V* maps (see Supplementary Figure 15). The defect does not seem centered around either an A, B, or C site, supporting the idea that the Se atom is positioned at a random lateral position in the van der Waals gap. The features in the d*I*/d*V* spectrum of the Se_i_ are not shifted with respect to those in a spectrum of a defect-free area (see Supplementary Figure 9e), even though Se_i_ is reported to be an *n*-type defect.^38^ The absence of a shift can be explained by the tip being too far away from the defect to measure its local doping effect.

Generally, a random distribution of defects is observed in single crystals or films grown epitaxially due to homogeneous and well-controlled growth conditions in processes such as MBE.^38,40^ Interestingly, for the wet-chemically prepared Bi_2_Se_3_ nanoplatelets, we observe clusters of defects of the same type. For example, in Figure 3g,h, we observed multiple Bi_Se(1)_ close together, suggesting a local excess of Bi. In contrast, in the area shown in Figure 3h, multiple Se_Bi(2)_ are observed, indicative of a local Se abundance. Usually, we see a large variety in defect density for different regions on various nanoplatelets, i.e., some areas contain many defects or a local cluster of defects, while others are almost completely defect-free (see Supporting Information). Although statistics are inherently limited in scanning probe studies, we usually observe a local abundance of one type of defect. We attribute these findings to the colloidal synthesis conditions, where local inhomogeneities in conditions, e.g., precursor concentration and temperature, can stimulate the local formation of defects and, more specifically, a particular type of defect.

Next, we hypothesize the implications of various defects on the optical properties of a single nanoplatelet. For five out of seven defects, the d*I*/d*V* spectra remain largely unchanged, except for a small shift to positive or negative energies in the local density of states, corresponding to the *n*- or *p*-type character of the defect (see Figure 3d,f,h–j). Hence, these defects do not introduce additional low-lying energy states and are therefore not expected to significantly affect the optical properties of a nanoplatelet. In contrast, for X_Se(1)_ and Bi_Se(1)_, we observe additional resonances close to the Fermi level (see Figure 3e,g). Therefore, we hypothesize that these defects are expected to significantly impact the optical properties, e.g., enabling carrier trapping or low-energy absorption.^43^ A direct relationship between the atomic structure and optical properties will require STM and optical spectroscopy on the same individual nanocrystal—a technically very demanding task that will be addressed in future studies.

Finally, we consider adsorbates on the nanoplatelet surface and their influence on the local electronic structure of the nanoplatelet. Figure 4a shows a part of a four-quintuple-layer nanoplatelet that is partly covered with adsorbates. Adsorbate-covered areas are recognized as cloudy structures, while the areas in between them are atomically clean. Multiple d*I*/d*V* spectra were measured on an adsorbate-covered and atomically clean area, as presented by the red and blue curves in Figure 4b, respectively, and as a contour plot in Figure 4c. The d*I*/d*V* spectra on both areas qualitatively show the standard electronic structure for a four-quintuple-layer nanoplatelet (see Figure 2d). However, there is a significant quantitative difference between the spectra of both areas: On average, the spectra taken on the adsorbate-covered area are shifted –93 mV relative to the atomically clean area. We attribute the change to a lower work function induced by the local dipole of the adsorbates.^44^ These findings highlight the importance of the clean transfer of nanocrystals onto a substrate. For applications where nanocrystals are used in dried form, ensuring surface cleanliness is crucial for maintaining the local electronic properties of the nanocrystal.

**Figure 4:**
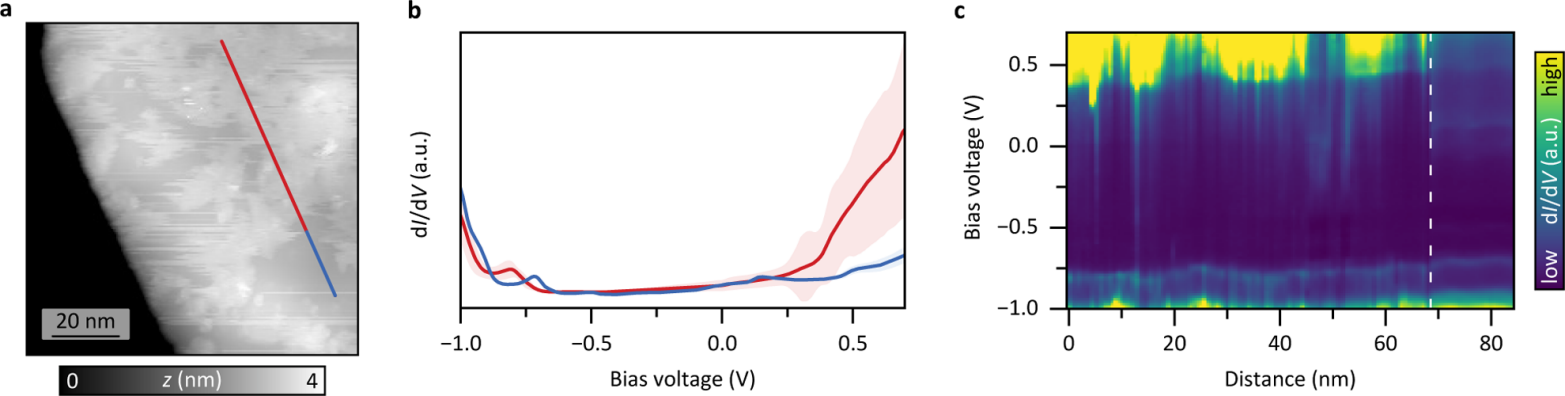
**Adsorbate-induced shift of the density of states. a**, STM image of a part of a four-quintuple-layer nanoplatelet on Au(111) (*V*_bias_ = –1 V, *I*_set_ = 50 pA). Background correction (plane leveling) is applied. Some parts of the nanoplatelet are covered with adsorbates, which appear as cloudy structures. In between these cloudy structures, the areas are atomically clean. Horizontal stripes are a result of tip-induced manipulation of adsorbates in the horizontal scan direction. **b**, Average of 80 (red) and 20 (blue) d*I*/d*V* spectra taken along an 84 nm line on the nanoplatelet depicted in (a). The gloom represents the standard deviation. The red and blue spectra are taken on adsorbate-covered and adsorbate-free areas, respectively. **c**, Contour plot of the d*I*/d*V* spectra shown in (b). The white dashed line indicates the boundary between adsorbate-covered (left) and adsorbate-free, i.e., atomically clean, (right) area.

In summary, using a multistep transfer technique, we deposited wet-chemically prepared nanocrystals onto an atomically clean Au(111) surface under ultrahigh vacuum conditions. The transfer technique, named viscoelastic nanoparticle transfer (VENT), largely avoids the detrimental cotransfer of ligands and solvent molecules, and thus enables atomic resolution imaging of the surface of nanocrystals. With Bi_2_Se_3_ nanoplatelets as an example, we image the atomic structure of the top surface, characterize surface and subsurface point defects, and their immediate impact on the electronic band structure with the same precision as for *in situ* ultrahigh vacuum-prepared samples. We find that certain defect types in wet-chemically prepared nanocrystals are not uniformly distributed, demonstrating local inhomogeneity during synthesis. In addition, we show that an adsorbate layer on the nanoplatelet surface results in a negative shift in the local potential, highlighting the importance of the clean transfer of nanocrystals. These findings demonstrate the valuable insights gained from simultaneous atomic resolution imaging and electronic characterization of single nanocrystals.

## Supplementary Material



## Notes

Comparison between VENT method and drop-casting; STM height traces of a four-quintuple-layer nanoplatelet; Influence of strain; Surface cleanliness of Au(111); More details on the defect analysis; Photograph of the transfer setup (PDF)
