# Supplementary material for: Simultaneous Atomic Resolution Imaging and Electronic Characterization of Wet-Chemically Prepared Nanocrystals
Source: Nano Lett. 2026 Jul 10;26(28):9163–9. doi: 10.1021/acs.nanolett.6c01858 (PMC13397881; doi:10.1021/acs.nanolett.6c01858)
Supplement: Supplementary file 1 [file nl6c01858_si_001.pdf]

Simultaneous atomic resolution imaging and electronic characterization of wet-chemically prepared nanocrystals (Supplementary Information)

Auke Vlasblom[1], Victor Wesselingh[1], Jara Vliem[1], Daniel Vanmaekelbergh[1], Ingmar Swart[1*]

[1]Debye Institute for Nanomaterials Science, Faculty of Science, Utrecht University, Princetonplein 1, Utrecht, 3584 CC, The Netherlands.

*Corresponding author(s). E-mail(s): i.swart@uu.nl

Methods

The VENT technique for the transfer of Bi_2Se_3 nanoplatelets onto Au(111) is illustrated in Figure 1a,b. To start, a 1x1 cm piece of polydimethylsiloxane (PDMS) is cut from a gel film (Gel-Pak® DGL-45x45-0065-X0). After, (1) a glass slide (2x2 cm) and the piece of PDMS are plasma-treated in air (0.4 mbar) at 40 kHz, 50 W for one minute. Directly after, (2) the plasma-treated sides are brought into contact, resulting in strong adhesion between the glass and PDMS.[1] Next, (3) the top surface of the PDMS is plasma-treated to remove residue[2] and increase the wettability of the PDMS surface.[3] Following, (4–6) a droplet (10 µL) of Bi_2Se_3 nanoplatelets diluted in ethanol is drop-cast on the PDMS, which is left to dry for two minutes. The Bi_2Se_3 nanoplatelets are stored in a N_2 glovebox and are transferred into the ambient atmosphere right before drop-casting. The volume of the droplet and concentration of nanoplatelets in ethanol are optimized such that the yield is at least one nanoplatelet per μm^2 (see Supplementary Note 1). The resulting glass slide–PDMS stamp is (7) glued with silver paint onto a small stainless steel block. The silver paint was left to dry for ~5 minutes. After, the stainless steel block is mounted on a linear arm that is placed in the (pressurized) load lock chamber of our vacuum setup. In total, the dried stamp is exposed to air for 10–15 minutes before the turbomolecular pump of the load lock chamber are turned on. The load lock chamber is then pumped down for 12–24 hours to a pressure of 4×10^{-9} mbar (the base pressure in the load lock is not affected by the presence of the PDMS stamp), after which (8) a vacuum-prepared Au(111) crystal is positioned in front of the linear arm. The linear shift (on which the stamp is placed) has only one degree of freedom: towards and away from the Au(111) sample. The Au(111) sample is placed in a rotatable magnetic transfer arm with a port aligner, which allows for the parallel positioning of the Au(111) sample and stamp to ensure there is a large contact area between the stamp and Au(111) sample. The linear shift and rotatable magnetic transfer arm were vertically aligned during assembly of the linear arm. Lastly, (9–10) the stamp is pushed to the Au(111) sample, resulting in the mechanical transfer of the nanoplatelets from the stamp to the Au(111) surface. A picture of the stamp setup in the vacuum chamber is provided in Supplementary Figure 17. Prior to STM measurements, the stamped Au(111) sample was annealed at 100 °C for 16 hours.

For the transfer of Bi_2Se_3 nanoplatelets onto mica, the procedure is similar to that for transferring Bi_2Se_3 nanoplatelets to Au(111). Steps 1–6 in Figure 1a,b are the same. After the PDMS stamp with dried Bi_2Se_3 nanoplatelets is fabricated, the stamp is placed (with the PDMS side facing down) on top of a freshly cleaved mica crystal that is lying flat (horizontal). The stamp is gently pressed onto the mica crystal using the thumb. After, the stamp is carefully taken off the mica crystal, making sure that there is no lateral movement of the stamp with respect to the mica. Directly after transfer, the mica crystal is placed under an ambient AFM.

Bi_2Se_3 nanoplatelets were synthesized as described elsewhere.[4]

A clean and flat mica substrate was obtained by cleaving a bulk mica crystal with scotch tape. An atomically clean Au(111) surface was obtained by repeated Ar^+ sputter (1 kV, 3×10^{-6} mbar) and anneal (450–500 °C) cycles in an ultra-high vacuum chamber (base pressure of $\sim 5 \times 10^{-10}$ mbar).

A Diener Zepto plasma cleaner operating at 40 kHz, 50 W and 0.4 mbar air pressure was used for plasma treatments of the glass slides and PDMS. AFM measurements were performed on a JPK Nanowizard II in intermittent-contact mode under ambient conditions. For imaging, Bruker OTESPA-R3 (26 N/m) SPM tips were used. For STM/STS measurements, we used an Omicron LT-STM operating at a temperature of 4.3 K and pressure in the 1×10^{-11} mbar range. The bias is applied to the sample. A Pt-Ir tip prepared on atomically clean Au(111) was used for all experiments. Spectroscopic data is obtained with an EG&G Instruments 7260 DSP lock-in amplifier, using a voltage bias modulation of 973 Hz. Lock-in amplitudes vary between of 5–10 mV rms. The dI/dV curves contain 201 or 301 datapoints, resulting in a spectral resolution between 6.6–10 mV. STM and AFM images are analyzed with Gwyddion 2.62.

Supplementary Note 1

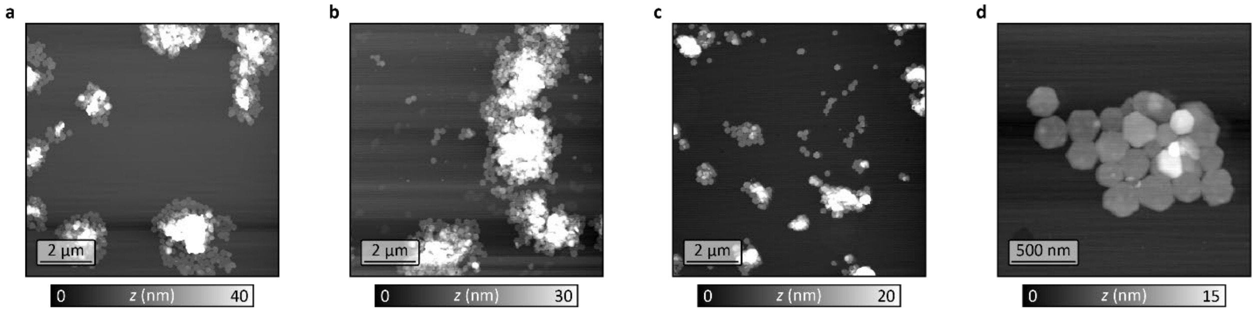

Supplementary Figure 1 | Drop-casting Bi_2Se_3 nanoplatelets on mica. a–c, AFM images of Bi_2Se_3 nanoplatelets drop-cast directly on a freshly cleaved mica substrate under ambient conditions. **d**, Close-up AFM image of one of the smaller stacks displayed in (c). Background correction (plane levelling) is applied to all images. Drop-casting results in large stacks (>40 nm) and an inhomogeneous distribution of nanoplatelets on the substrate. Because of clustering and stacking, individual nanoplatelets are observed only occasionally.

To compare the VENT technique with a conventional technique, i.e., drop-casting, a 10 µL droplet of Bi_2Se_3 nanoplatelets diluted in ethanol was drop-cast on mica and the distribution and coverage of the nanoplatelets were analysed with ambient AFM (see Supplementary Figure 1). Mica was used as a test substrate because large, flat, and clean terraces are obtained by cleaving with scotch tape, making the material suitable for the deposition of nanoplatelets and AFM experiments. The AFM images after drop-casting reveal one of the most common issues after drop-casting: Nanoplatelets aggregate, resulting in large stacks and consequently, an inhomogeneous coverage. This issue leads to the observation that individual nanoplatelets are rarely observed. When the VENT technique is used with mica as the target substrate, this issue is solved (see Figure 1c), as will be briefly elucidated next.

The distribution of nanoplatelets takes place during drop-casting on PDMS and is determined by the wetting of the solvent on the PDMS.[3] Hence, the distribution of nanocrystals is not dependent on the target substrate, as is the case for conventional methods, e.g., drop-casting. Accordingly, the VENT technique will be able to provide a consistent distribution for various target substrates, provided an optimized drop-casting procedure on PDMS is available. The coverage can be tuned by changing the concentration of nanoplatelets and the volume of the droplet, as is standard procedure for drop-casting.

Before the VENT technique was used in ultra-high vacuum conditions for transferring Bi_2Se_3 nanoplatelets to Au(111), the technique was optimized for transferring Bi_2Se_3 nanoplatelets on mica under ambient conditions. We assumed that using the VENT technique on mica in air and on Au(111) in vacuum would produce a similar distribution and coverage of Bi_2Se_3 nanoplatelets on Au(111) as on mica, as this is mainly determined by the drop-casting step on PDMS (see previous paragraph). For the optimization, two goals were set: the nanoplatelet distribution must be homogeneous, i.e., the number of large stacks needs to be minimized (to avoid damage to the STM tip during scanning), and the coverage must be sufficient (around one isolated nanoplatelet per 1–4 μm^2). The goals were reached by using a plasma treatment on the PDMS before drop-casting, which improved the wetting of the PDMS.[3] Adding this crucial step resulted in a good distribution of nanoplatelets (also lowering the number of large stacks). The coverage was tuned by finding an appropriate dilution of nanoplatelet solution and droplet volume.

When following the steps of the VENT technique, it should be noted that during the mechanical transfer step (Figure 1b (9)), the nanocrystals are turned over. In other words, the side that was interfaced with the PDMS (the bottom surface) now becomes exposed to vacuum (the top surface). This means that the surface that is imaged is only exposed to the PDMS surface and not to air during the steps under ambient conditions. This will help in preventing oxidation of the surface and the adsorption of contaminants (present in the ambient atmosphere) on the surface of the nanocrystals. For nanocrystals that are extremely prone to oxidation, the steps that are performed in ambient atmosphere can, in principle, be carried out in an inert atmosphere. This would, however, complicate the procedure as a compatible protective suitcase is needed to transport the stamp to a vacuum setup.

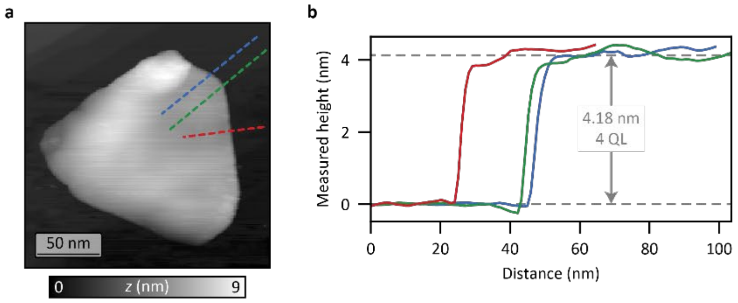

Supplementary Figure 2 | Measured height of a four-quintuple-layer Bi_2Se_3 nanoplatelet. a, STM image of the nanoplatelet shown in Figure **2**a (V_{bias} = 1 V, I_{set} = 50 pA). **b,** Three height traces of the surface of the nanoplatelet shown in (a), indicated by the (same coloured) dashed lines. The height traces are vertically aligned such that the Au(111) surface corresponds to a measured height of around 0 nm. The nanoplatelet is not entirely flat, and height traces are drawn such that flat regions of the nanoplatelet are considered. An average height of ~4.18 nm is found for the nanoplatelet, corresponding to four quintuple layers (one quintuple layer corresponds to 0.96 nm). Considering the presence of residue under the nanoplatelet, together with the corrugation of the nanoplatelet surface, we expect that the apparent height is an overestimation of the actual height of the nanoplatelet. Therefore, the number of quintuple layers is rounded down to the nearest integer: four quintuple layers.

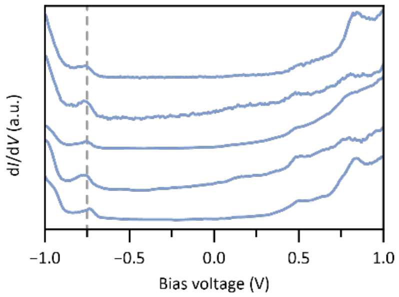

Supplementary Figure 3 | Differential conductance spectra on five different four-quintuple-layer Bi_2Se_3 nanoplatelets. d*I*/d*V* spectra measured on five different Bi_2Se_3 nanoplatelets with the same thickness (four quintuple layers). All spectra are taken on an atomically clean area on the nanoplatelet. The position of the prominent valence band peak varies between –740 mV and –767 mV; it is centered around –750 mV as indicated by the gray dashed line. The spectra are offset for clarity.

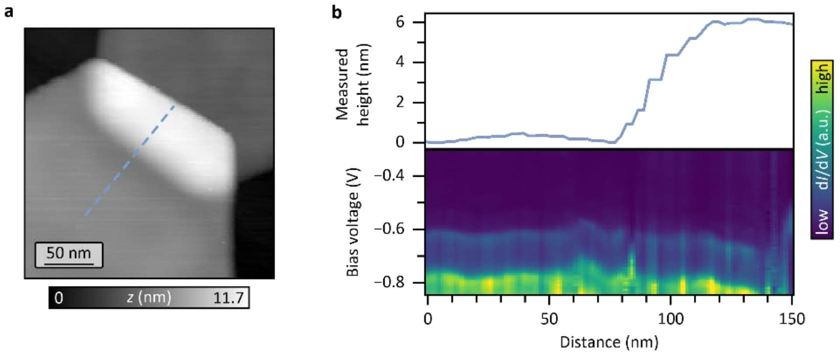

Supplementary Figure 4 | Influence of strain on the electronic structure. a, STM image of a nanoplatelet (bottom left) that is folded over another nanoplatelet (top right) (V_{bias} = –1 V, I_{set} = 30 pA). The shape of the bottom nanoplatelet is visible on the top nanoplatelet. **b,** Top: Height trace of the nanoplatelet over the folded area, indicated by the dashed blue line in (a). The large curvature of the nanoplatelet will induce strain in the top nanoplatelet. Bottom: 65 d*I*/d*V* spectra taken along the same dashed blue line indicated in (a). We do not observe any correlation between the local curvature and the energy of the prominent valence band peak, indicating that strain does not significantly influence the electronic structure.

Supplementary Note 2

Before performing the VENT technique, the Au(111) surface quality was assessed and was found to be atomically clean: atomic steps and the herringbone reconstruction are observed (see Supplementary Figure 5a). After performing the VENT technique, a sub-monolayer of contamination is observed on the Au(111) surface (see Supplementary Figure 5b), which we attribute to solvent molecules, excess precursor materials, ligands, and residue from the PDMS stamp. A smaller-scale image of the Au(111) surface is shown in Supplementary Figure 5c. Both figures (b and c) show that atomic features, i.e., step edges and herringbone reconstruction, are still observable, which is a significant improvement in cleanliness compared to an Au(111) surface after drop-casting.[4] A small-scale image of the contamination is given in Supplementary Figure 5d. At the bottom of the image, dotted assemblies are observed, which are reminiscent of excess precursor materials, such as Bi precursor. Next to dotted assemblies, stripes are recognized, which are reminiscent of the self-assembly of fatty acids, which might have adsorbed on the PDMS during the preparation steps under ambient conditions.

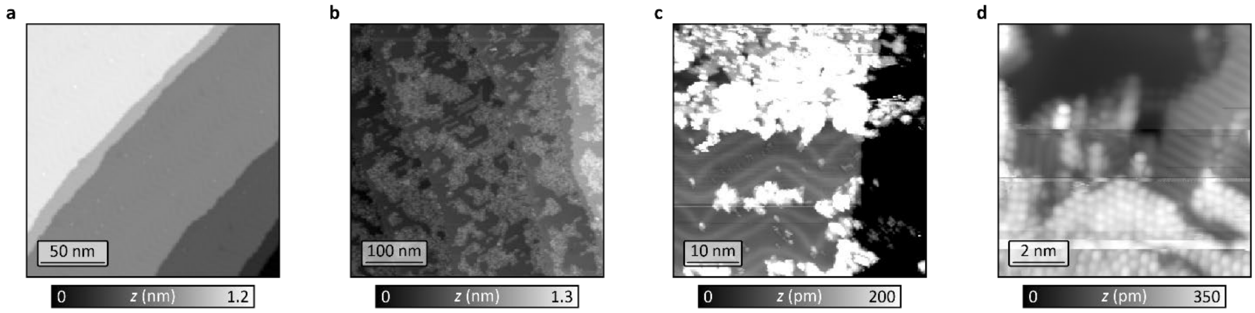

Supplementary Figure 5 | Cleanliness of the Au(111) surface after the VENT technique. a, STM image of a vacuum-cleaned Au(111) surface before the mechanical transfer of nanoplatelets (V_{bias} = –1 V, I_{set} = 1 nA). **b**–**d**, STM images of the Au(111) surface after performing the VENT technique. **b**, Large-scale STM image of the Au(111) surface (V_{bias} = 1 V, I_{set} = 50 pA). Atomic steps and a sub-monolayer coverage of contamination is visible. **c**, Small-scale STM image of the Au(111) surface after the VENT procedure (V_{bias} = –1 V, I_{set} = 30 pA). Despite contaminants, the herringbone reconstruction of the Au(111) surface remains visible. **d**, Close-up STM image of the contaminants on the Au(111) surface (V_{bias} = 1 V, I_{set} = 30 pA). Background correction (plane levelling) is applied to all images.

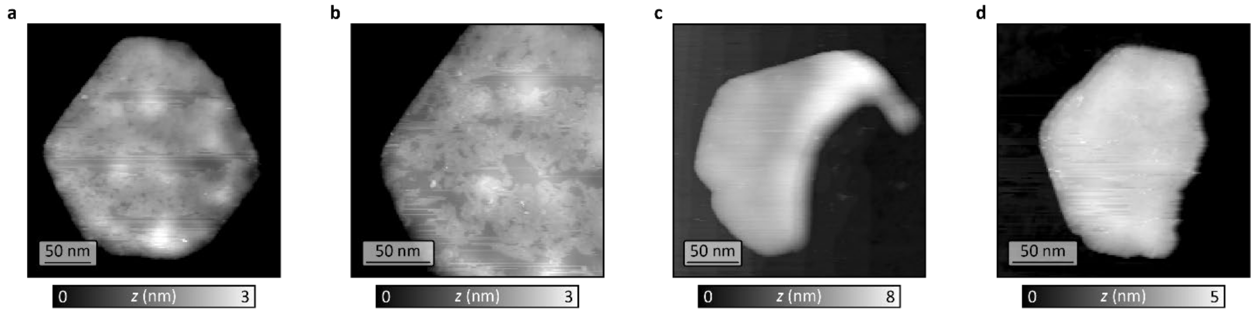

Supplementary Figure 6 | Cleanliness and damage of Bi_2Se_3 nanoplatelets. a, STM image of a Bi_2Se_3 nanoplatelet covered with a sub-monolayer of contamination (V_{bias} = –0.8 V, I_{set} = 50 pA). Horizontal stripes occur due to contamination being dragged by the STM tip during scanning. **b,** Close-up STM image of the nanoplatelet shown in (a) (V_{bias} = –1 V, I_{set} = 50 pA). Patches of contamination are recognized as cloudy structures. In between the contamination, the nanoplatelet surface is atomically clean. **c**, STM image of a broken nanoplatelet (V_{bias} = –1 V, I_{set} = 50 pA). **d**, STM image of a broken nanoplatelet (V_{bias} = 1 V, I_{set} = 20 pA). Background correction (plane levelling) is applied to all images.

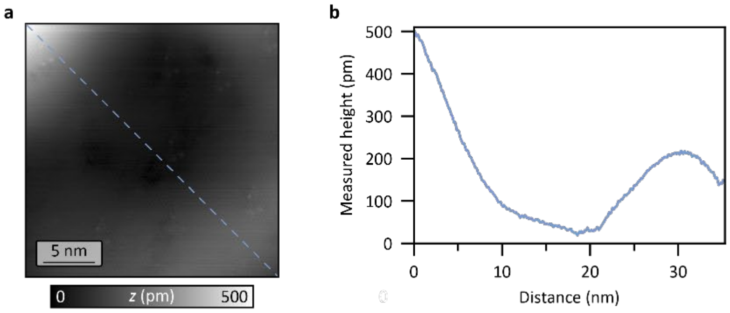

Supplementary Figure 7 | Corrugation on the surface of a Bi_2Se_3 nanoplatelet. a, STM image of the surface of the nanoplatelet shown in Figure **2**a (V_{bias} = 1 V, I_{set} = 50 pA). The image is the same as Figure **2**b, but here the background is plane-levelled. There is poor contrast of the atomic lattice due to large-scale corrugations on the nanoplatelet surface. **b**, Height trace of the surface of the nanoplatelet surface shown in (a), indicated by the dashed blue line. The corrugation of the nanoplatelet surface is larger than the corrugation of the atomic lattice.

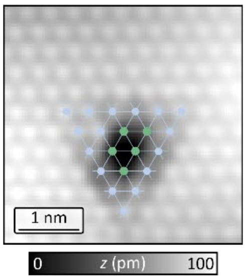

Supplementary Figure 8 | Analysis of multi-$v_{Se(1)}$. STM image shown in main Figure 3e with a schematic overlay of the Se(1) lattice (light blue circles and lines) (V_{bias} = –0.8 V, I_{set} = 100 pA). Five missing Se(1) atoms, corresponding to A sites, are indicated by green circles.

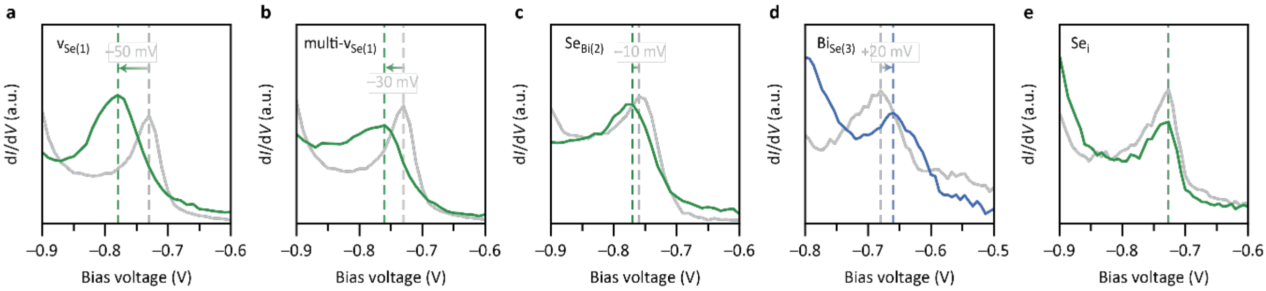

Supplementary Figure 9 | Zoom-ins of d*I*/d*V* spectra taken on defects. a–e, Zoom-ins on the prominent valence band peak of the d*I*/d*V* spectra shown in **Figure 3**d, e, h, i, and j, respectively. Dashed lines indicate the position of the prominent valence band peak for a d*I*/d*V* spectrum taken on a defect-free (grey curve) area and a defect (coloured curve). A $v_{Se(1)}$, multi-$v_{Se(1)}$, and a $Se_{Bi(2)}$ result in a negative spectral shift, while a $Bi_{Se(3)}$ results in a positive spectral shift. For a Se_i, no spectral shift is visible.

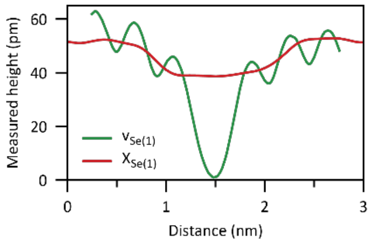

Supplementary Figure 10 | Height traces on $v_{Se(1)}$ and $X_{Se(1)}$. Height traces of a $v_{Se(1)}$ (green curve) shown in Figure 3d and of a $X_{Se(1)}$ (red curve) shown in Figure 3f. An $X_{Se(1)}$ appears as an indent at a Se(1) site, but the indent appears less deep than a $v_{Se(1)}$.

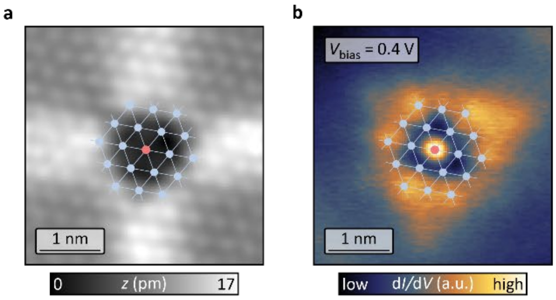

Supplementary Figure 11 | Analysis of $X_{Se(1)}$. a, STM image shown in main Figure 3f with a schematic overlay of the Se(1) lattice (light blue circles and lines) (V_{bias} = –0.8 V, I_{set} = 400 pA). The red circle indicates the indent on a Se(1) site. **b,** Constant height dI/dV map (V_{bias} = 0.4 V) taken on the same location as (a). The same schematic lattice overlay used in (a) is shown to indicate the Se(1) lattice. At the indented Se(1) site, indicated by the red circle, a high dI/dV intensity is observed that is localized at a single Se(1) site. A triangular symmetry is observed around the defect.

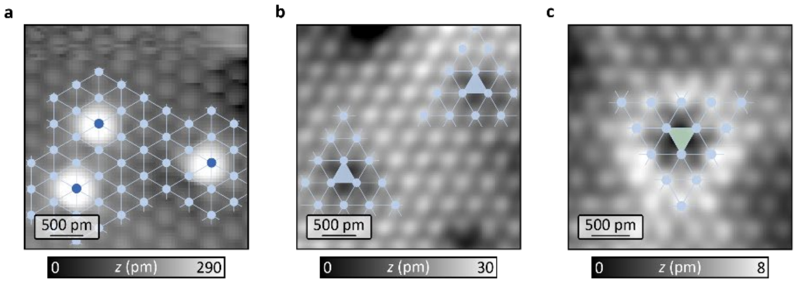

Supplementary Figure 12 | Analysis of anti-site defects. STM images shown in main Figure 3g-i with a schematic overlay of the Se(1) lattice (light blue circles and lines). **a**, Three $Bi_{Se(1)}$ are identified on Se(1) sites, indicated by dark blue circles. V_{bias} = –1 V, I_{set} = 50 pA. **b**, Two $Se_{Bi(2)}$ are identified on B sites, indicated by light blue triangles. V_{bias} = 1 V, I_{set} = 0.5 nA. **c**, A single $Bi_{Se(3)}$ is identified on a C site, indicated by the green triangle. V_{bias} = –1 V, I_{set} = 40 pA.

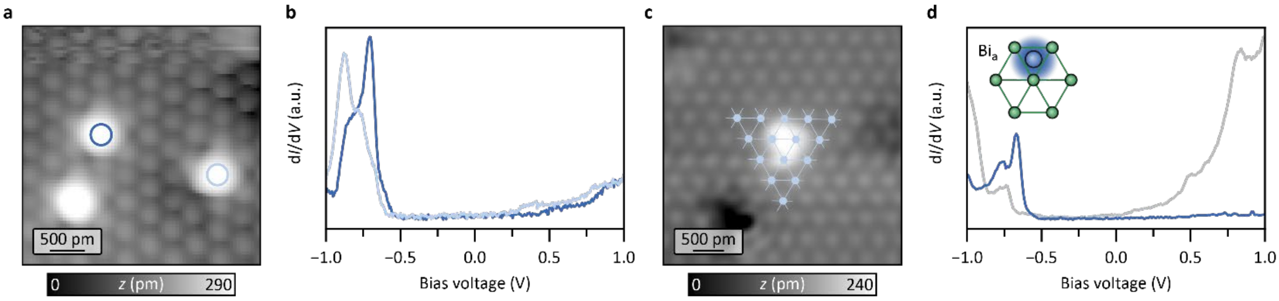

Supplementary Figure 13 | Electronic structure of two different $Bi_{Se(1)}$ anti-site defects and a Bi adsorbate. a, STM image shown in main Figure 3g (V_{bias} = –1 V, I_{set} = 50 pA). **b,** dI/dV spectra taken on two different $Bi_{Se(1)}$ as indicated in (a) by the dark and light blue circles. The two spectra show different resonances in the valence band. **c,** STM image of a Bi adsorbate (Bi_a) (V_{bias} = –1 V, I_{set} = 400 pA). The schematic Se(1) lattice (light blue circles and lines) reveals that the Bi_a is located between a hollow and bridge site. Also note the non-circular shape of the Bi_a species. **d,** dI/dV spectrum measured on Bi_a shown in (c) (blue curve) and on a defect-free area (grey curve). The spectrum on Bi_a is similar to the spectra measured on $Bi_{Se(1)}$ (panel b).

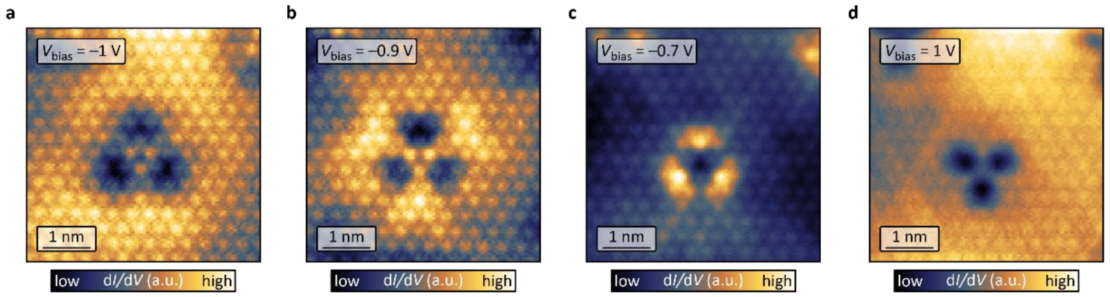

Supplementary Figure 14 | Constant height d*I*/d*V* maps of a $Bi_{Se(3)}$ anti-site defect. a-d, Constant height d*I*/d*V* maps measured on a $Bi_{Se(3)}$ at various bias voltages as indicated in the images. A clover-leaf symmetry can be recognized, reminiscent of a $Bi_{Se(3)}$. The electronic contrast changes for different bias voltages.

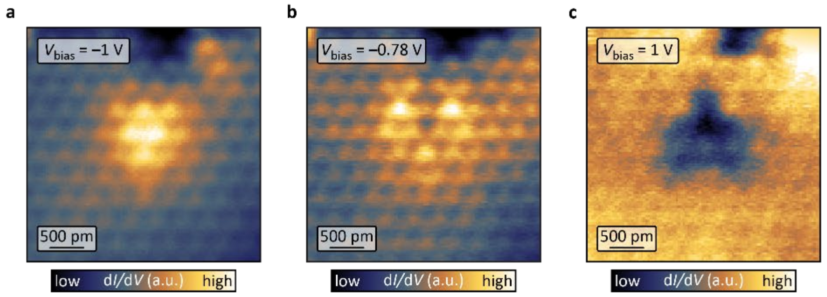

Supplementary Figure 15 | Constant height d*I*/d*V* maps of a Se_i defect. a-c, Constant height d*I*/d*V* maps measured on a Se_i at various bias voltages as indicated in the images. A triangular symmetry is recognized. The center of the triangle does not correspond to an A, B or C site, but is localized on a bridge site.

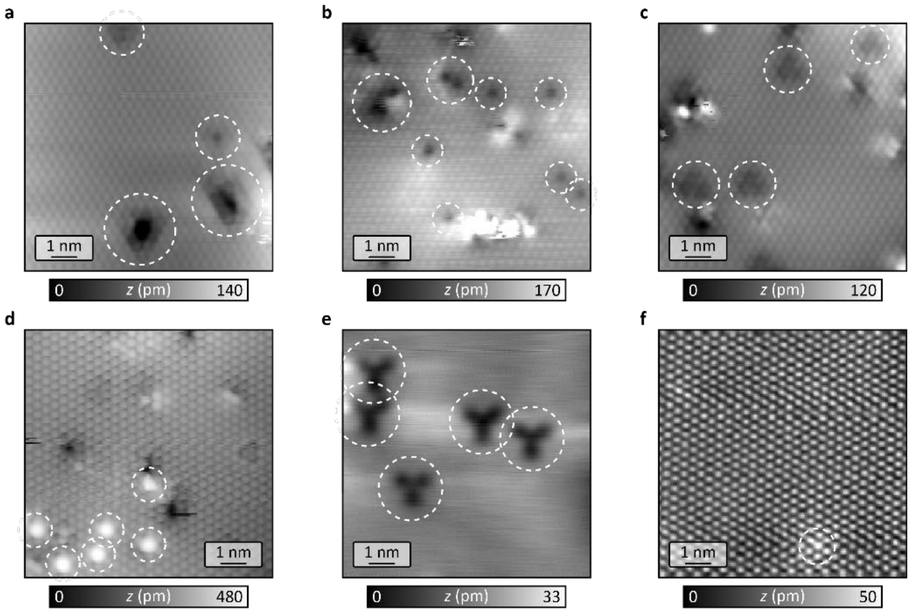

Supplementary Figure 16 | Clustering of defects. a-f, STM images of different areas on various nanoplatelets. All images are the same size (100 nm^2). Background correction (plane level or polynomial) is applied to all images. A 2D FFT filter is applied to the image in (f). White dashed circles highlight (some) of the most abundant defects in the image. Defect densities varies between different areas. Often, a local abundance of one specific type is defect is observed. **a**, Area with only (multi-)$v_{Se(1)}$. V_{bias} = –0.8 V, I_{set} = 100 pA. **b**, Apart from some adsorbates, this area contains many $v_{Se(1)}$. V_{bias} = –1 V, I_{set} = 50 pA. **c**, This area contains many defect. The most abundant defect is $Se_{Bi(2)}$. V_{bias} = –1 V, I_{set} = 100 pA. **d**, Area with a large variety in defect types. At the bottom left, there is a local cluster of $Bi_{Se(1)}$. V_{bias} = –1 V, I_{set} = 50 pA. **e**, Area with only $Bi_{Se(3)}$. V_{bias} = 1 V, I_{set} = 30 pA. **f**, This area is almost completely defect-free. One sub-surface defect is seen at the bottom of the image. V_{bias} = –1 V, I_{set} = 1 nA.

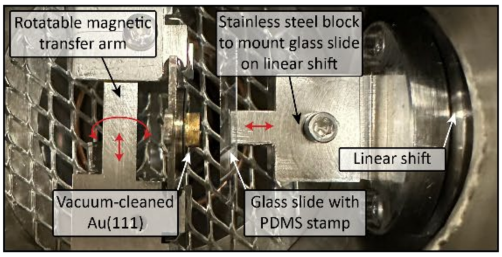

Supplementary Figure 17 | Photo of the transfer setup in the vacuum chamber. The glass slide, containing a PDMS stamp with dried Bi_2Se_3 nanoplatelets, is glued to a stainless steel block. This block is mounted on a linear shift, which can move linearly towards and away from the Au(111) sample. The vacuum-cleaned Au(111) sample is placed in a rotatable magnetic transfer arm, which is used to position the Au(111) directly in front of the stamp. A port aligner on the transfer arm (not in the photo) can be used to finetune the alignment between the Au(111) sample and the stamp.

References

1. Chau, K. *et al.* Dependence of the quality of adhesion between poly(dimethylsiloxane) and glass surfaces on the composition of the oxidizing plasma. *Microfluid. Nanofluidics* **10**, 907–917 (2011).
2. Jain, A. *et al.* Minimizing residues and strain in 2D materials transferred from PDMS. *Nanotechnology* **29**, 265203 (2018).
3. Chen, I. J. & Lindner, E. The stability of radio-frequency plasma-treated polydimethylsiloxane surfaces. *Langmuir* **23**, 3118–3122 (2007).
4. Moes, J. R. *et al.* Characterization of the Edge States in Colloidal Bi2Se3 Platelets. *Nano Lett.* **24**, 5110–5116 (2024).
